# The impact of fetuin-A on predicting aortic arch calcification: secondary analysis of a community-based survey

**DOI:** 10.3389/fcvm.2024.1415438

**Published:** 2024-07-08

**Authors:** Yi-Hung Lin, Meng-Hung Lin, Chung-Sheng Shi, Yu-Sheng Lin, Chun-Liang Lin, Yao-Hsu Yang, Yu-San Liao, Mei-Yen Chen, Ming-Horng Tsai, Ming-Shyan Lin

**Affiliations:** ^1^ Division of Cardiology, Department of Internal Medicine, Chang Gung Memorial Hospital Chiayi Branch, Chiayi, Taiwan; ^2^ Health Information and Epidemiology Laboratory, Chang Gung Memorial Hospital Chiayi Branch, Chiayi, Taiwan; ^3^ Graduate Institute of Clinical Medical Sciences, College of Medicine, Chang Gung University, Taoyuan, Taiwan; ^4^ Division of Nephrology, Department of Internal Medicine, Chang Gung Memorial Hospital Chiayi Branch, Chiayi, Taiwan; ^5^ Department of Traditional Chinese Medicine, Chang Gung Memorial Hospital Chiayi Branch, Chiayi, Taiwan; ^6^ School of Traditional Chinese Medicine, College of Medicine, Chang Gung University, Taoyuan, Taiwan; ^7^ Department of Diagnostic Radiology, Chang Gung Memorial Hospital, Chiayi, Taiwan; ^8^ Department of Nursing, Chang Gung University of Science and Technology, Chiayi, Taiwan; ^9^ Department of Nursing, Chang Gung University, Taoyuan, Taiwan; ^10^ Department of Pediatrics, Chang Gung Memorial Hospital, Yunlin, Taiwan

**Keywords:** atherosclerosis, aortic arch, calcification, fetuin-A, hypertension

## Abstract

**Introduction:**

Atherosclerotic cardiovascular disease is associated with a high mortality rate due to vascular calcification. The role of fetuin-A in aortic arch calcification (AAC) is less well understood.

**Methods:**

An analysis of secondary biomarkers was performed on 800 individuals from the biobank using the community database. AAC was defined by radiologists based on imaging. Multiple variables logical analysis was used for risk analysis.

**Results:**

A total of 736 individual samples were collected based on age and gender. The average age is 65 ± 10 years, and half the population comprises men. In spite of similar body weight, renal function, and hepatic function, the AAC group had higher blood pressure and fetuin-A levels independently: systolic blood pressure (SBP) index ≥130 mmHg [adjusted odds ratio (aOR) 1.85, 95% confidence interval (CI) 1.34–2.57, *p *= 0.002] and fetuin-A (aOR 0.62, 95% CI 0.50–0.76, *p *< 0.001). Moreover, it is evident that AAC can be predicted more accurately when combined with SBP ≥130 mmHg and a low fetuin-A level (<358 μg/ml: aOR 5.39, 95% CI 3.21–9.08) compared with the reference.

**Conclusion:**

Low fetuin-A levels are significantly correlated with AAC while there is an increased association between vascular calcification and coexisting hypertension.

## Introduction

Atherosclerotic cardiovascular disease (ASCVD) has emerged as a major health issue worldwide, resulting in high mortality rates and preventive efforts (1). A phenotype of ASCVD that poses a significant challenge is vascular calcification, which can cause critical stenosis and complicate intervention. Vascular calcification is an important indicator for estimating cardiovascular disease, and it also serves as a prognostic factor, especially for extracoronary calcification, including carotid and thoracic aortic calcification (2–4). In contrast to costly or invasive coronary artery calcification imaging, the detection of aortic arch calcification (AAC) through chest x-rays (CXRs) is feasible and accessible during an annual checkup using a semi-quantitative method, while it could be strictly classified by an aortic computed tomography (CT)-based Agatston score (4–6). Bodies of studies have shown that patients with AAC are at high risk of further cardiovascular events and all-cause mortality (3, 4, 6). Early identification and investigation of pathogenic mechanisms are essential for preventing poor vascular and survival outcomes.

Beyond many mechanisms contributing to vascular calcification, the fetuin-A also has been identified as circulating calcification inhibitors and potential biomarkers of vascular calcification (7–9). In addition to having multifunctional roles on the mineral gatherer, it also binds calcium phosphate mineral (CaP) in calciprotein particles (CPPs) and prevents crystal growth and maturation (10, 11). Furthermore, fetuin-A levels have been shown to be inversely correlated with microvascular complications, plaque burden, carotid atherosclerotic progression, coronary artery calcification, valvular or abdominal aortic calcification, thoracic aortic aneurysm, cardiovascular events, and mortality (12–17). Most studies focus on chronic kidney disease or dialytic populations, coronary artery, or carotid artery as well as abdominal aorta; however, the association between fetuin-A and AAC has been less examined (3, 9, 14–16). Despite the fact that oxidative stress also contributes to vascular calcification, fetuin-A may modulate oxidative stress to some extent, especially in obesity, although the exact mechanisms involved in vascular calcification remain to be determined (9, 18).

Therefore, we investigate biomarkers such as fetuin-A in secondary samples collected from participants in community health checkups. The purpose of our study is to determine whether there are any risk factors or protective factors that might contribute to the development of AAC. In addition, the effects of fetuin-A coexisting oxidative stress and hypertension were examined in AAC patients.

## Methods

### Population and study design

**Figure 1:**
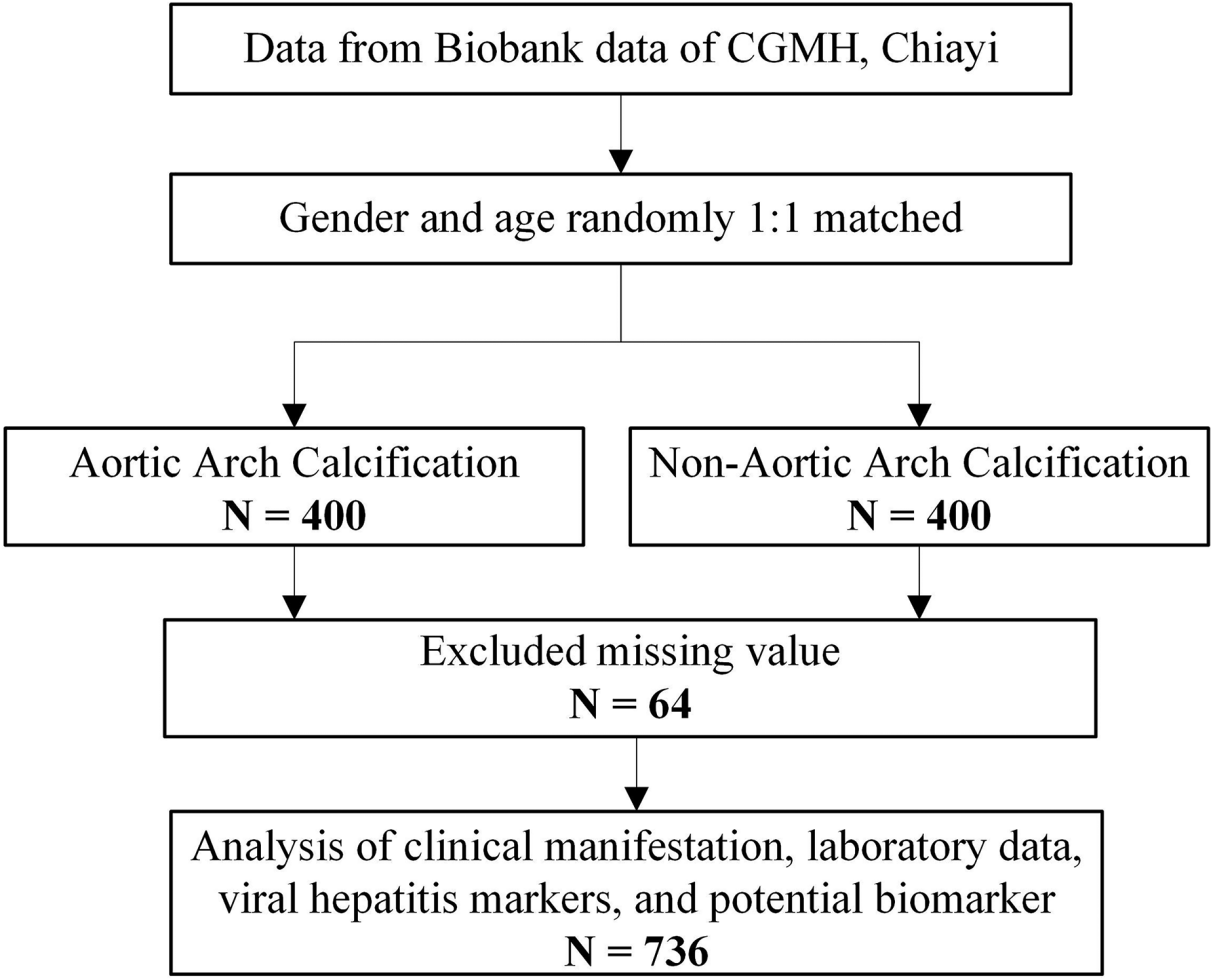
Enrollment of study subjects.

This cross-sectional study was conducted among adult patients in southern Taiwan who attended annual checkups between 2017 and 2019. Our study collected data on the patients’ anthropometric measures, laboratory results, and hepatitis virus markers. The participants completed an informed consent form and a questionnaire. Serum samples were stored in the biobank, Chang Gung Memorial Hospital (CGMH), Chiayi. A total of 800 samples were enrolled for further biomarkers analysis based on the matching of sex and age (Figure 1). After excluding participants with incomplete data, 736 participants were included in the final analysis (Figure 1). The Institutional Review Board and Ethics Committee of Chang Gung Memorial Hospital approved this study (IRB No. 202101596B0).

### Anthropometric measurements

All participants in this study underwent two measurements of blood pressure using an electronic sphygmomanometer [OMRON HEM-1000, 6 MCN0937000, OMRON (Dalian) Co., Ltd, Dalian, China] in a seated position following a period of 10 min of rest. This study was conducted to record the mean arterial pressure (systolic and diastolic). To estimate body mass index (BMI), each participant’s standing height and weight were measured to 0.1 cm and 0.1 kg, respectively, while wearing light clothing and without shoes.

### Laboratory analysis

A sample of blood was collected from a patient after fasting for 12 h and tested in the laboratory of the CGMH Hospital. A series of biochemical tests were performed (Roche Diagnostics, Cobas6000, C501, Mannheim, Germany) on serum creatinine (Cr), uric acid, sugar, aspartate aminotransferase (AST), alanine aminotransferase (ALT), triglyceride (TG), low density lipoprotein cholesterol (LDL-C), high density lipoprotein cholesterol (HDL-C), total cholesterol (TC), and apolipoprotein B (Apo-B). Hemoglobin (Hb) and platelet (PLT) count check-ups are performed using the XN-3000 (Sysmex Taiwan Co., Ltd., Taipei, Taiwan). Standard ELISA was used to detect HB surface antigen (HBsAg) (General Biological Corp., Hsinchu, Taiwan), and SP-NANBASE C-96 3.0 plate was used to detect anti-Hepatitis C virus (HCV) antibodies (General Biological Corp., Hsinchu, Taiwan).

### Biomarkers and oxidative stress markers

Serum levels of fetuin-A and interleukin-6 (IL-6) were determined by an ELISA method (Elabscience, Houston, TX, USA). Spot urine samples were collected and stored below 80 °C until they could be quantified in the central laboratory of the collaborating hospital. A competitive enzyme-linked immunosorbent assay kit (ELISA; Japan Institute for the Control of Aging, Fukuroi, Japan) was used to measure urinary 8-hydroxy-2-deoxyguanosine (8-OHdG). The concentration of 8-OHdG was adjusted for urinary creatinine and expressed as ng/mg creatinine.

### Definition of AAC

Following the manufacturer’s instructions, the participants were scanned by a diagnostic imaging technician at the hospital, and they were given a plain film for posterior–anterior chest roentgenography (SHIMADZU, Model 0. 6/1. 2P364DK-85. Kyoto, Japan). Vascular calcification was defined as AAC grade 0–3 divided into four levels: grade 0, no visible calcification; grade 1, a few thin spots of calcification; grade 2, a thick calcification; grade 3, circular calcification of the aortic knob (5–8). According to the interpretive reports of two clinicians, grades 1–3 were assigned to the AAC group, whereas those without detectable calcification (grade 0) were assigned to the non-AAC group. The acquisition of AAC grading is illustrated in Supplementary Figure S1.

### Statistical analysis

The demographic characteristics of the participants according to AAC statuses (AAC vs. none) were compared using the chi-square test for categorical variables and the independent sample t-test for continuous variables. Using the demographics/characteristics as explanatory variables, a series of univariate conditional logistic regression analyses were performed to initially screen the potentially associated factors of AAC. The multivariable conditional logistic regression model was used to compute odds ratios (ORs) with 95% confidence intervals (CIs) for AAC adjusted for potential risk factors including BMI, systolic blood pressure (SBP), and laboratory data after matching by age and gender. Finally, a receiver operating characteristic (ROC) curve analysis was conducted to evaluate the ability of the fetuin-A and SBP >130 mmHg to discriminate the presence of AAC. All tests were two-tailed, and a *p*-value <0.05 was considered signiﬁcant. Data analyses were performed using SAS version 9.4 (SAS Inc., Cary, NC, USA).

## Result

### Participants’ basic characteristics as determined by AAC

A total of 736 participants (grade 1 = 315, grade 2 = 36, grade 3 = 17 in AAC, *n* = 368, vs. non-AAC, *n* = 368) who completed the community health screening were included in the final analysis. Based on age, gender distributions, body weight, and BMI, there is no significant difference between the patients in the AAC and non-AAC groups (Table 1). The average age of the participants was 65 ± 10 years, and nearly half of them were men. Both groups have similar body weight, BMI, and renal and hepatic functions, as well as Hb and PLT count. In addition, they also had similar lipid profiles for LDL-C, HDL-C, cholesterol, triglycerides, Apo-B, uric acid, and fasting sugar levels, whereas 8-OHdG, IL-6, and viral markers were not significantly different in comparison. Furthermore, we found that fetuin-A levels were significantly lower in the AAC group compared with none (388.3 ± 127.9 vs. 438.3 ± 131.1, *p* < 0.001). In addition, the median value and range of fetuin-A among the different estimated glomerular filtration rate (eGFR) categories is not significant (Supplementary Tables S1, S2).

**Table 1:** Baseline characteristics of participants according to aortic arch calcification status.

Variables	AAC	Non-AAC	*p-*value
Total, *n*	368	368	
Age (years), mean ± SD	65.7 ± 10.0	65.3 ± 10.0	0.641
Age (years), *n* (%)			1.000
20–40	6 (1.6)	6 (1.6)	
41–60	101 (27.5)	101 (27.5)	
61–80	235 (63.9)	235 (63.9)	
≥80	26 (7.1)	26 (7.1)	
Sex, *n* (%)			1.000
Male	185 (50.3)	185 (50.3)	
Female	183 (49.7)	183 (49.7)	
Body weight (kg), mean ± SD	66.7 ± 12.5	66.0 ± 11.7	0.441
BMI (kg/m^2^), mean ± SD	26.0 ± 3.9	25.7 ± 3.5	0.262
SBP (mmHg), *n* (%)			<0.001
<130	183 (49.7)	244 (66.3)	
≥130	185 (50.3)	124 (33.7)	
Laboratory data, mean ± SD			
Hb (g/dl)	14.0 ± 1.5	14.0 ± 1.6	0.785
PLT (10^3 ^/μl)	223.8 ± 60.8	230.7 ± 63.9	0.135
Cr (mg/dl)	0.9 ± 0.5	1.0 ± 0.5	0.721
eGFR (ml/min/1.73 m^2^)	83.2 ± 23.7	81.5 ± 22.3	0.331
TC (mg/dl)	185.8 ± 40.6	183.2 ± 38.1	0.357
LDL-C (mg/dl)	117.5 ± 35.2	119.4 ± 36.2	0.491
TG (mg/dl)	123.7 ± 73.9	122.8 ± 69.7	0.856
HDL-C (mg/dl)	51.0 ± 13.5	52.3 ± 13.0	0.202
Apo-B (mg/dl)	100.1 ± 25.3	98.6 ± 25.9	0.418
ALT (U/L)	31.1 ± 32.4	32.1 ± 36.7	0.705
AST (U/L)	27.0 ± 18.2	26.1 ± 24.3	0.559
Uric acid (mg/dl)	5.9 ± 1.6	6.0 ± 1.5	0.669
Fasting sugar (mg/dl)	111.4 ± 33.6	112.2 ± 36.1	0.752
Viral hepatitis markers			
HBsAg seropositive, *n* (%)	63 (17.1)	65 (17.7)	0.846
Anti-HCV seropositive, *n* (%)	66 (17.9)	83 (22.6)	0.119
Potential biomarkers (mean ± SD)			
8-OHdG (ng/mg creatinine)	42.0 ± 19.2	44.1 ± 22.8	0.169
Fetuin-A (μg/ml)	388.6 ± 128.0	434.4 ± 107.6	<0.001
IL-6 (pg/ml)	3.9 ± 11.2	3.6 ± 8.4	0.659

### Univariate and multivariate data analysis

**Figure 2:**
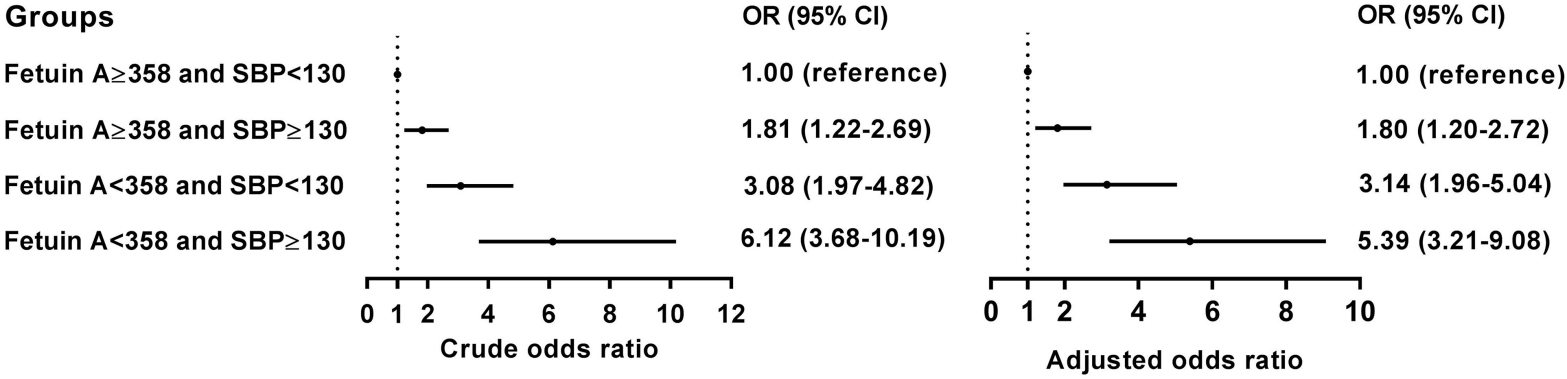
Conditional logistic analysis for AAC according to the cutoff values for the SBP and the fetuin-A.

Upon analyzing multivariate data, the risk of developing AAC in patients with an SBP index ≥130 mmHg (aOR 1.85, 95% confidence interval 1.34–2.57, *p *= 0.002), while fetuin-A seems to serve as a factor against AAC (aOR 0.62, 95% confidence interval 0.50–0.76, *p *< 0.001) after adjustment for potential risk factors including BMI, SBP, and laboratory data following matching by age and gender (Table 2). Accordingly, we analyzed the receiver operating characteristic curve for these arms to determine the area under the ROC curve (AUC), which is 0.648 and the minimum cutoff value is 358 μg/mg for fetuin-A (Supplementary Figure S2). To test the interaction of SBP and fetuin-A on incidental AAC, we found that coexisting SBP  ≥130 mmHg and fetuin-A ≤358 μg/ml had predicted the highest risk of incidental AAC (hazard ratio 5.39, 95% CI 3.21–9.08, *p* < 0.001) when compared with SBP with lower levels and fetuin-A with higher levels (Figure 2).

**Table 2:** Conditional logistic regression analysis of the association between aortic arch calcification and laboratory data.

Variable	Crude		Adjusted^a^	
	OR (95% CI)	*p*-value	OR (95% CI)	*p*-value
BMI (kg/m^2^)	1.11 (0.92–1.34)	0.249	1.05 (0.85–1.29)	0.633
SBP (mmHg)				
<130	Ref.	—	Ref.	—
≥130	1.93 (1.43–2.61)	<0.001	1.85 (1.34–2.55)	<0.001
Laboratory data				
eGFR (ml/min/1.73 m^2^)	1.12 (0.91–1.38)	0.272	1.12 (0.89–1.41)	0.315
TG (mg/dl)	1.01 (0.88–1.16)	0.855	0.92 (0.77–1.09)	0.352
HDL-C (mg/dl)	0.86 (0.70–1.06)	0.165	0.84 (0.65–1.08)	0.178
Apo-B (mg/dl)	1.09 (0.90–1.32)	0.371	1.10 (0.88–1.38)	0.360
8-OHdG (ng/mg creatinine)	0.89 (0.75–1.05)	0.181	0.90 (0.75–1.07)	0.253
Fetuin-A (μg/ml)	0.60 (0.49–0.73)	<0.001	0.59 (0.48–0.72)	<0.001
IL-6 (pg/ml)	1.00 (0.97–1.04)	0.664	1.00 (0.96–1.04)	0.831

^a^ The model was adjusted for BMI, SBP, eGFR, TG, HDL, Apo-B, 8-OHdG, fetuin-A, and IL-6 after matching by age and gender. The odds ratios calculated for an interquartile range increase in BMI and laboratory data.

### The association of fetuin-A and AAC in diverse oxidative stress distribution

**Figure 3:**
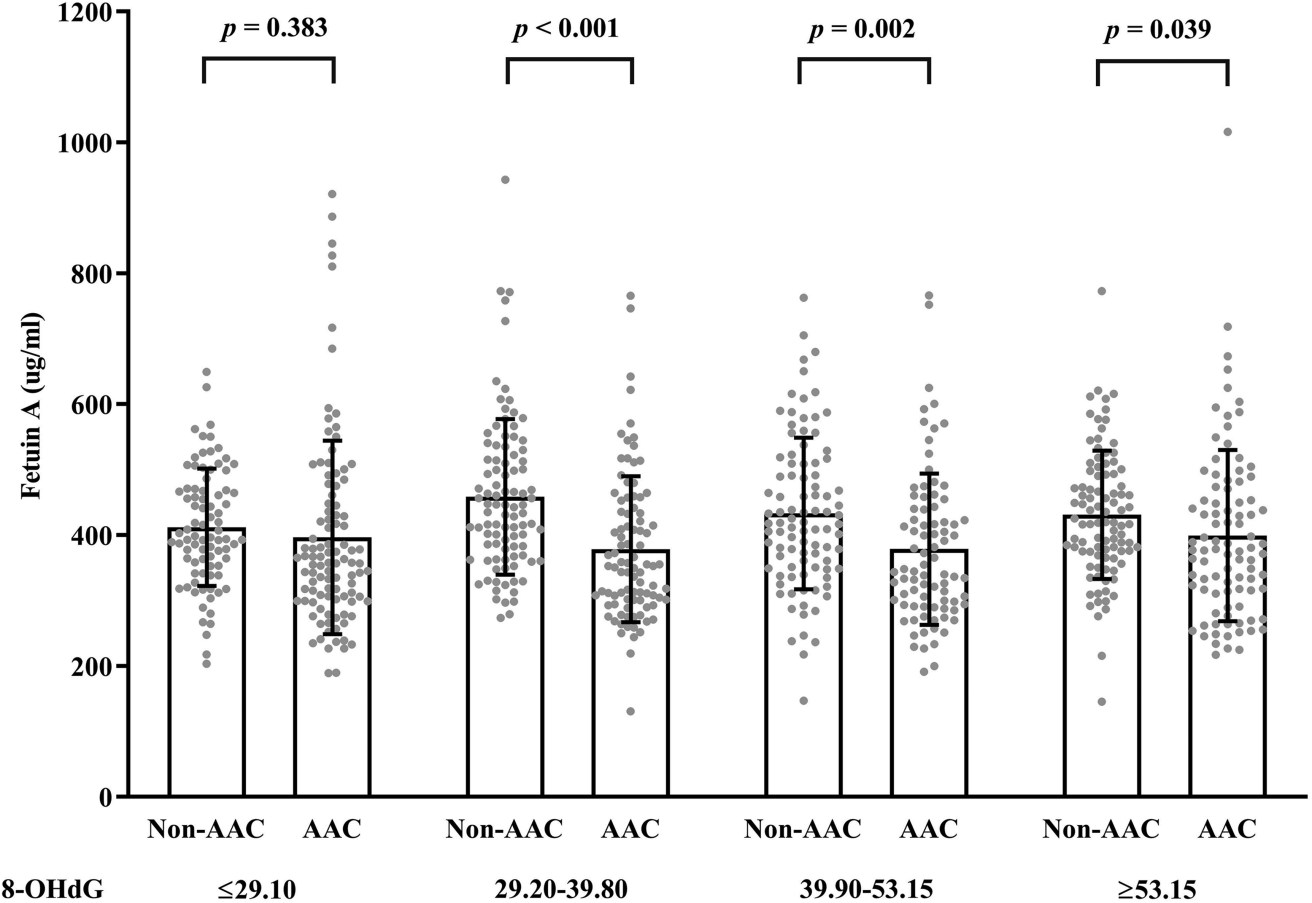
Scatter bar chart of fetuin-A levels across non-AAC and AAC groups with different 8-OHdG levels.

Figure 3 illustrates the relationship between fetuin-A levels, AAC, and 8-OHdG levels. The graph divides subjects into four groups based on the interquartile range of 8-OHdG levels: ≤29.10, 29.20–39.80, 39.90–53.15, and ≥53.15. When comparing individuals with and without AAC, fetuin-A levels tend to be lower, particularly when using the higher ranges of 8-OHdG levels (>29.10). There is a clear distinction between the AAC and non-AAC groups as 8-OHdG levels increase due to statistically significant differences (*p*-values < 0.05).

### Development of a predicting nomogram

**Figure 4:**
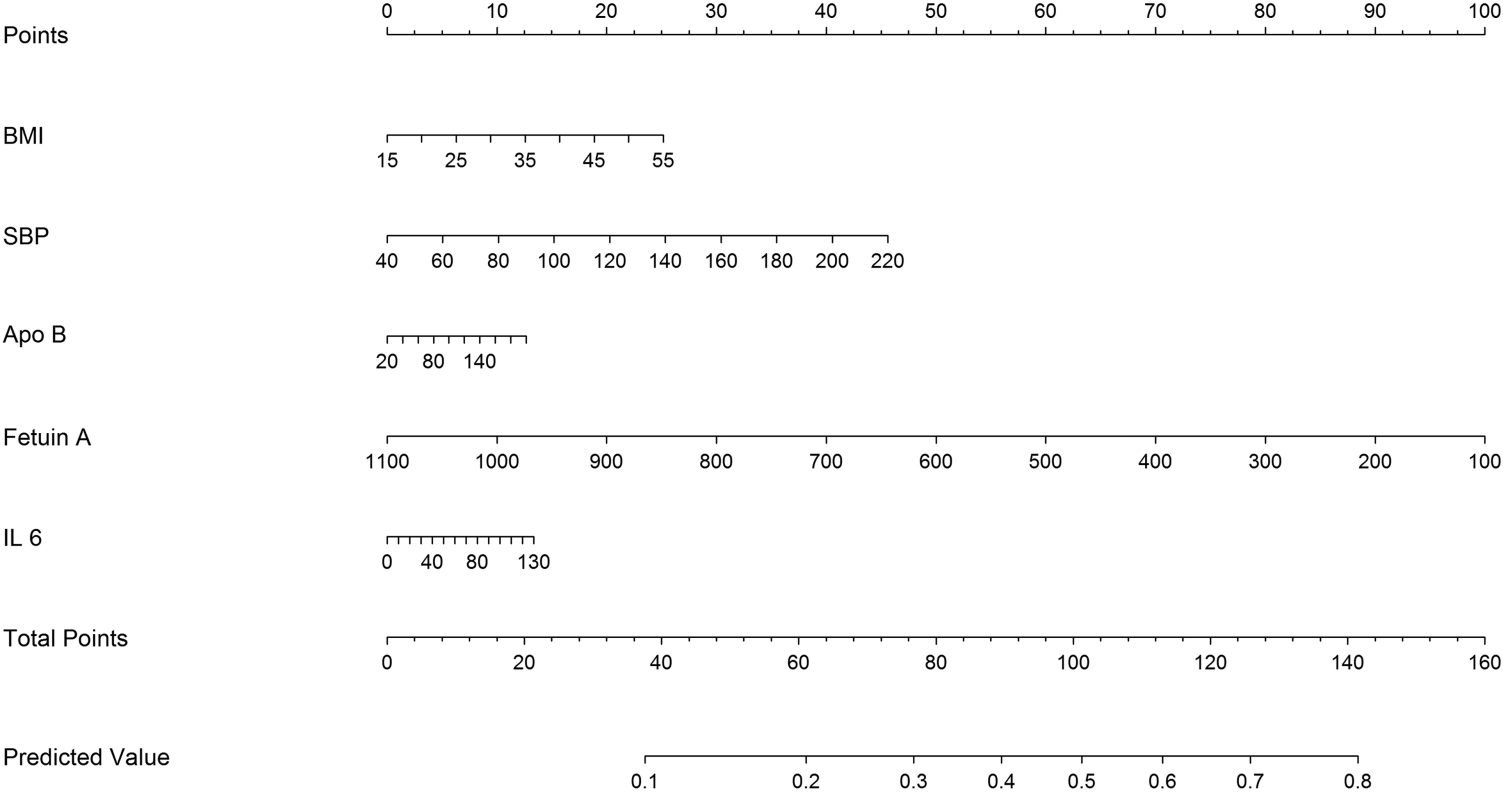
Development of a predictive nomogram for predicting AAC probability.

Considering the prognostic signiﬁcance of the fetuin-A biomarker, we sought to combine it with nine common clinical factors to better predict the presence of AAC. Through univariate logistic regression we examined the prognostic signiﬁcance of the two biomarkers and three clinical factors, including BMI, Apo-B, SBP, fetuin-A, and IL-6 level. Fetuin-A and SBP status are the major components of the clinical characteristics for AAC (Figure 4).

## Discussion

To investigate potential predictive markers for AAC risk, a secondary analysis was conducted on a retrospective community database. During routine physical examinations, chest x-ray could detect AAC that was inversely correlated with fetuin-A. In hypertensive populations with SBP greater than 130 mmHg, the combination of lower fetuin-A levels may have a synergistic effect that may increase the risk of AAC. The results also provide a greater understanding of the pathophysiology and further disease detection.

According to community health examinations, AAC can play a critical role in the risk stratification of cardiovascular disease burden by using a simple but crucial method (3). AAC may result in an increase in cardiovascular mortality as well as worsening the progression of ASCVD (12–17, 19). Apart from well-known risk factors, AAC is mainly caused by aging and hypertension, with high blood pressure causing endothelium dysfunction and systemic inflammation (19–21). Our findings showed SBP ≥130 mmHg was correlated independently with incidental AAC, indicating calcification of the arterial walls to deter high resistance.

Beyond high BP ≥130 mmHg, we further discovered that the fetuin-A level has an inverse correlationship with AAC, which showed diverse effects at different stages of diseases (22). Detecting significant reductions in the serum fetuin-A is a predicting marker for vascular calcification (9, 17, 23). According to the STRAMBO study, lower levels of fetuin-A are associated with severe abdominal aorta calcification, but the study was restricted to men over 40 years of age, and the focus was on the abdominal aorta (20). Likewise, another study found a significant negative correlation between fetuin-A levels and the degree of abdominal aortic calcification among stone farmers (24). Chen et al. concluded that patients with lower fetuin-A showed higher percentages of AAC on a plain chest x-ray defined posterior–anterior profile compared with patients with higher fetuin-A (25). However, the study population consisted of dialytic patients, and the number of participants was small. Until recently, few studies have been conducted on the causal relationship between AAC and fetuin-A in the general population, whereas our results suggest fetuin-A plays a negative role in vascular calcification as AAC.

Moreover, we found that coexisting hypertension (HTN) (SBP ≥ 130 mmHg) and lower fetuin-A had a greater additive effect than a single predictor: aOR = 5.39 over the single predictor for incidental AAC (Figure 2). The STRAMBO study also reported the coexistence of low fetuin-A and hypertension increases the odds of severe abdominal aortic calcification (20). Eleftheriadou et al. found that in patients with type 2 diabetes mellitus (T2DM), the odds of peripheral arterial disease increased with hypertension, dyslipidemia, increasing diabetes duration, smoking, and lower levels of fetuin-A (26). Specifically, low fetuin-A levels are associated with increased carotid artery intima media thickness as well as local arterial stiffness in children with hypertension (27). The fetuin-A gene was an essential predictor of atherosclerosis and vascular damage, whereas the study completed the puzzle of arterial wall calcification. Based on our findings in Table 2, we developed a predicting nomogram that included potential risks that can be used to predict AAC (Figure 4).

There is evidence that oxidative stress and inflammation may play a role in vascular calcification, which revealed similar levels of 8-OHdG and IL-6 in an AAC group compared with a non-AAC group (28). In spite of the inverse relationship between fetuin-A and 8-OHdG in obesity, our study concluded that fetuin-A still consistently predicts AAC risk among subjects with higher 8-OHdG levels, which may contribute to the progression of atherosclerotic disease (18). Therefore, fetuin-A may be a potential predictor of vascular calcification in addition to oxidative stress markers and traditional risk factors.

## Limitation

It is important to note that this retrospective study still contains biases and limitations. As part of this community study, only 368 AAC patients were matched with non-AAC patients. To enhance the power of these markers, it may be necessary to include a larger scale and longitudinal cohort. Standardizing the evaluation of SBP in a controlled environment is not possible, and only mean SBP can be computed, which may result in a false reading for patients with fluctuating SBP. As a rough examination, a CXR cannot distinguish between intimal and medial calcification. Therefore, observer bias cannot be completely eliminated. As a result of the limited data available, smoking or other habits were not included in our study, but no direct association was observed in the STRAMBO study for a group of smokers (20). The consumption of tobacco was found to be negatively associated with fetuin-A concentrations, which may contribute to the development of vascular calcification after smoking has been adjusted for. Consequently, fetuin-A proteins play a very important role in the stratification of AAC risks beyond traditional risks, even ignoring them in the case of AAC. Furthermore, many confounding factors including medications and comorbidities such as diabetes, dyslipidemia, and chronic kidney disease may influence the fetuin-A levels. To minimize confounding effects, we examined the distribution across the different eGFR categories (Supplementary Tables S1, S2) and completed lipid profiles and fasting sugar profiles.

## Conclusion

As is currently known that hypertension is associated with AAC, our study found that AAC was independently affected by a lower level of fetuin-A, which appears to exert anti-calcification effects. Furthermore, lower fetuin-A levels combined with a higher SBP may be able to provide enhanced overall predictive power for AAC. We need to conduct further large-scale and longitudinal studies to better understand the relationship between fetuin-A and AAC and their pathophysiology.

## Data Availability

The original contributions presented in the study are included in the article/**Supplementary Material**, further inquiries can be directed to the corresponding author.
