# Supplementary material for: The impact of fetuin-A on predicting aortic arch calcification: secondary analysis of a community-based survey
Source: Front Cardiovasc Med. 2024 Jul 8;11:1415438. doi: 10.3389/fcvm.2024.1415438 (PMC11260669; doi:10.3389/fcvm.2024.1415438)

Supplementary Table 1. Median with range for fetuin-A by AAC type.

| eGFR ml/min/1.73m^2^ | AAC | Non–AAC | Total |
| --- | --- | --- | --- |
| ≤29 | 310.7 (262.1–484.1) | 421.6 (362.4–528.9) | 393.2 (262.1–528.9) |
| 30–44 | 498.8 (248.9–827.2) | 382.6 (279.4–528.2) | 406.1 (248.9–827.2) |
| 45–59 | 353.1 (227.1–592.9) | 392.4 (273.4–592.9) | 369.2 (227.1–592.9) |
| ≥60 | 360.1 (131.0–1016.5) | 427.7 (145.4–943.2) | 396.9 (131.0–1016.5) |

Abbreviations: AAC, aortic arch calcification; eGFR, estimated glomerular filtration rate.

Supplementary Table 2. Median with Q1–Q3 for fetuin-A by AAC type.

| eGFR ml/min/1.73m2 | AAC | Non–AAC | Total |
| --- | --- | --- | --- |
| ≤29 | 310.7 (265.5–370.5) | 421.6 (393.2–507.4) | 393.2 (310.7–484.1) |
| 30–44 | 498.8 (363.9–545.3) | 382.6 (336.6–419.8) | 406.1 (363.9–528.2) |
| 45–59 | 353.1 (311.0–416.8) | 392.4 (344.6–476.8) | 369.2 (327.7–446.9) |
| ≥60 | 360.1 (300.1–448.3) | 427.7 (364.7–504.1) | 396.9 (323.6–475.1) |

Abbreviations: AAC, aortic arch calcification; eGFR, estimated glomerular filtration rate.

Supplementary figure 1 Image showing aortic arch calcification and its distribution in grades on an X-ray of the chest.


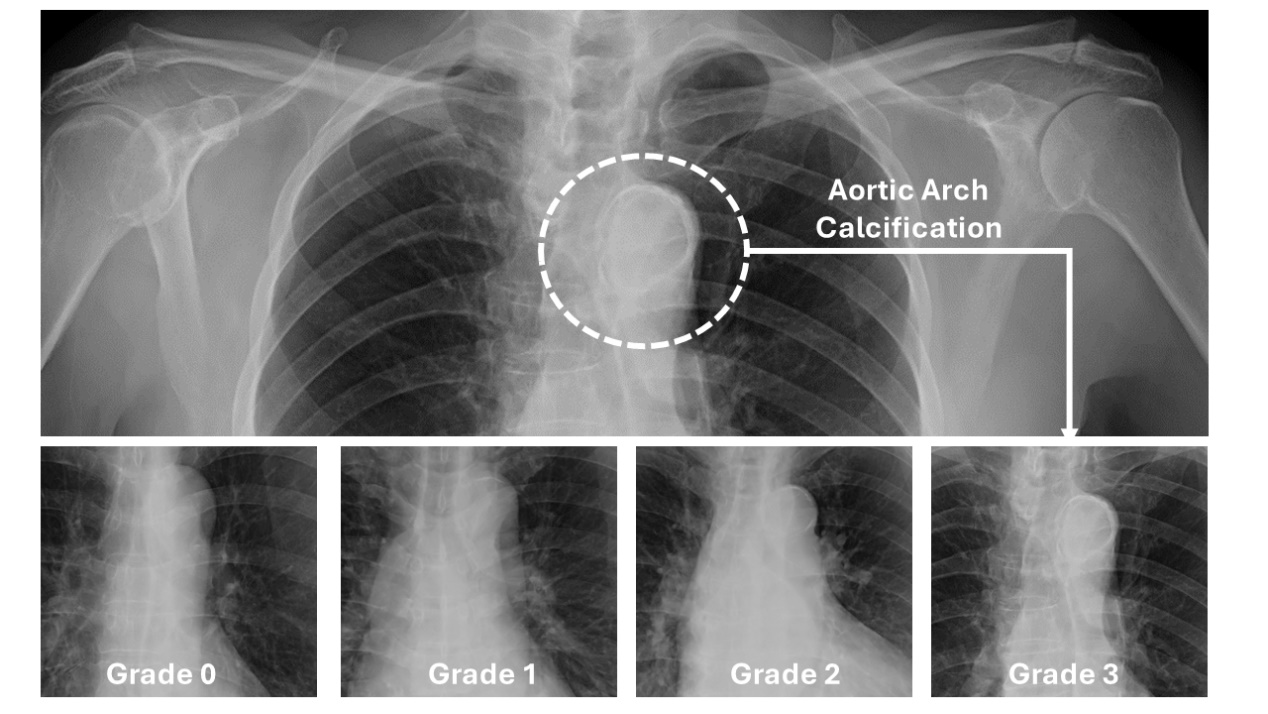


Supplementary figure 2 The ROC curve (AUC) and the minimum cut-off value for fetuin-A.


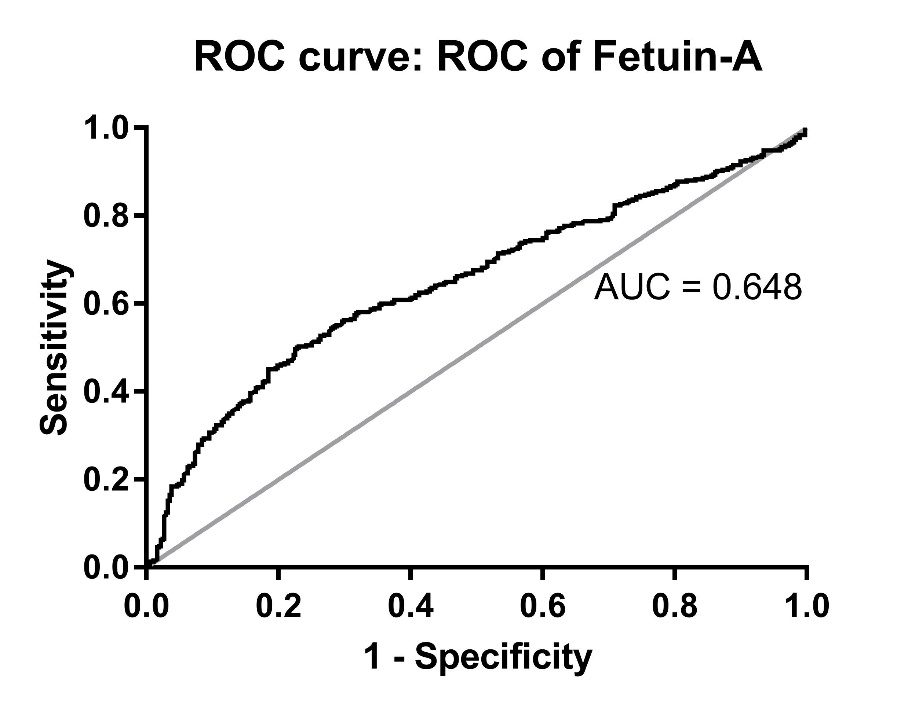

Supplement: Supplementary file 1 [file Datasheet1.docx]
